# β4-integrin-mediated cytotoxic activity of AexU in human prostate cancer PC3 cells

**DOI:** 10.3892/ol.2013.1542

**Published:** 2013-08-23

**Authors:** MASAFUMI KUMANO, HIDEAKI MIYAKE, SAID K. ABOLGHAIT, HOSNY M. BEHNSAWY, MASATO FUJISAWA

**Affiliations:** 1 Division of Urology, Kobe University Graduate School of Medicine, Kobe, Japan; 2 Faculty of Veterinary Medicine, Suez Canal University, Ismailia, Egypt; 3 Department of Urology, Faculty of Medicine, Assiut University, Assiut

**Keywords:** prostate cancer, AexU, β4-integrin, antitumor effect

## Abstract

The present study aimed to characterize the cytotoxic activity of AexU, an effector-mediating type three secretion system (TTSS) of gram-negative bacteria, in human prostate cancer cells, focusing on the association with β4-integrin expression. The cytotoxic effects of AexU either alone or in combination with chemotherapeutic agents were evaluated using several human prostate cancer cell lines. Human prostate cancer PC3 cells, in which an expression vector containing siRNA targeting β4-integrin had been introduced, were established (PC3/sh-In), and the cytotoxic effects of AexU on the PC3/sh-In cells were compared with the PC3 cells that were transfected with a control vector (PC3/C). The expression levels of β4-integrin in the PC3 cells were markedly higher compared with those in the LNCaP or DU145 cells, and the cytotoxic effects of AexU in the PC3 cells were more pronounced compared with those in the LNCaP or DU145 cells. The sensitivity of the PC3 cells to docetaxel and cisplatin was significantly enhanced following treatment with AexU, resulting in a decrease in the IC_50_ of the two agents by ~90%. The cytotoxic effect of AexU in the PC3/C cells was more marked compared with that in the PC3/sh-In cells, and the phosphorylation of Akt in the PC3/C cells appeared to be significantly more inhibited by the treatment with AexU compared with the PC3/sh-In cells. In conclusion, treatment with AexU may be a useful therapeutic option for prostate cancer when β4-integrin is overexpressed. The treatment appears to exert its effects through growth inhibition and by enhancing the sensitivity of the cancer cells to chemotherapeutic agents.

## Introduction

Numerous types of gram-negative bacteria possess the type III secretion system (TTSS), which delivers bacterial proteins from the bacterial outer membrane into the host. The bacterial proteins that influence the host physiology by translocation into the host cells via the TTSS are generally called ‘effectors’ (1). A TTSS effector, AexT, with homology to *Pseudomonas aeruginosa* bifunctional toxins, ExoT/S, was originally identified from a fish pathogen, *Aeromonas salmonicida*(2), while AexU, an AexT-like TTSS effector, has more recently been characterized from the small subunit (SSU) of *Aeromonas hydrophila*(3). The AexU gene is 1,539 bp in length, encodes a protein with 512 amino acid residues and has 38% identity with the AexT gene over the entire length (3). Although the activity of adenosine diphosphate (ADP)-ribosyltransferase in AexU has been well documented (4), AexU has also been shown to possess guanosine triphosphate (GTP)ase-activating protein (GAP) activity, which is mainly responsible for host cell apoptosis and the disruption of actin filaments (5). However, the cytotoxic impact of AexU on host cells and its molecular mechanism remain largely unknown.

The integrins, a family of transmembrane heterodimers, function as receptors for extracellular matrix molecules and mediate signal transduction to control a wide variety of cell functions, including survival, proliferation, angiogenesis and migration (6). Furthermore, the aberrant expression of integrins has been shown to play significant roles in the acquisition of an aggressive phenotype in cancer cells (7–9). In our previous study, the cytotoxic effect of AexU was shown to be exaggerated in the presence of increased β4-integrin expression (10), thus suggesting a significant role for the association between AexU and β4-integrin and indicating that AexU may be applicable to cancer therapy.

To date, several studies have reported a significant role for β4-integrin in the regulation of the malignant phenotype of various types of cancer cells, including prostate cancer (11–15). Drake *et al*(13) reported that the upregulation of β4-integrin that is induced by the stable knockdown of ZEB1, a major regulator of the epithelial-mesenchymal transition, resulted in a significant enhancement of the invasive potential of human prostate cancer cells. Based on these previous findings, the cytotoxicity of AexU was evaluated in the present study either alone or in combination with chemotherapeutic agents against human prostate cancer PC3 cells, and the mechanism underlying AexU-induced antitumor activity was analyzed, focusing on the significance of β4-integrin protein expression.

## Materials and methods

### Tumor cell lines

PC3, LNCaP and DU145 cells, derived from human prostate cancer, were purchased from the American Type Culture Collection (ATCC; Rockville, MD, USA). The cell lines were maintained in RPMI-1640 (Life Technologies, Inc., Gaithersburg, MD, USA) supplemented with 5% heat-inactivated fetal bovine serum.

### Preparation of AexU protein

The AexU protein was produced as described previously (10). Briefly, the AexU gene was amplified using primers, including a C-terminal hemagglutinin (HA) epitope tag. The gene was then sub-cloned into a *Bam*HI and *Sal*I-digested pGEX-5X-1 vector (GE Healthcare, Hino, Japan) and the AexU protein was produced as a recombinant glutathione-S-transferase-AexU-HA fusion protein.

### Expression plasmid and transfection into PC3 cells

A chemically synthesized oligonucleotide encoding a β4-integrin short hairpin RNA (shRNA) with a loop motif was inserted downstream of the U6 promoter of the pBAsi hU6 Pur DNA vector (Takara Bio, Inc., Otsu, Japan). The sequence of the shRNA targeting β4-integrin was 5′-CTACGACAGCTT CCTTATGTA-3′. Similarly, a control plasmid was constructed by randomizing the sequence of the shRNA (5′-GGAATCTCA TTCGATGCATAC-3′).

The expression vectors were transfected into the PC3 cells by liposome-mediated gene transfer methods, as described previously (16). Briefly, either the purified expression plasmid that contained the shRNA targeting β4-integrin or the control plasmid was added to the PC3 cells following pre-incubation for 30 min with Lipofectamine™ 2000 (Invitrogen, San Diego, CA, USA) and serum-free Opti-MEM^®^ (Life Technologies, Inc.). Drug selection in 1.5 μg/ml Puromycin (Sigma, St. Louis, MO, USA) was performed for three days following transfection and the colonies were harvested and expanded to generate the cell lines.

### In vitro cell growth assay

To evaluate the cytotoxicity of AexU on the *in vitro* proliferation of the tumor cell lines, 5×10^4^ cells from each cell line were seeded into 12-well plates and the number of cells was assessed daily in triplicate using Cell Counting kit-8 (Dojindo Molecular Technologies, Inc., Kumamoto, Japan). In addition, the effects of the treatment with cisplatin or docetaxel (Sigma) in combination with AexU on the proliferation of the PC3 cells were also examined.

### Western blot analysis

The western blot analyses were performed as described previously (17). In brief, samples that contained equal amounts of protein (25 μg) from the lysates of the cultured tumor cell lines were analyzed using antibodies against total and phosphorylated Akt, β4-integrin, cleaved caspase-3 (Cell Signaling Technology, Inc., Danvers, MA, USA), Bax, β-actin (Santa Cruz Biotechnology, Inc., Santa Cruz, CA, USA) and horseradish peroxidase-conjugated secondary antibodies (Santa Cruz Biotechnology, Inc.).

### Statistical analysis

The differences between the two groups were compared using unpaired t-tests. All the statistical calculations were performed using the StatView 5.0 software program (Abacus Concepts, Inc., Berkeley, CA, USA). P<0.05 was considered to indicate a statistically significant difference.

## Results

### Cytotoxic effects of AexU on prostate cancer cells depends on the expression of β4-integrin

**Figure 1:**
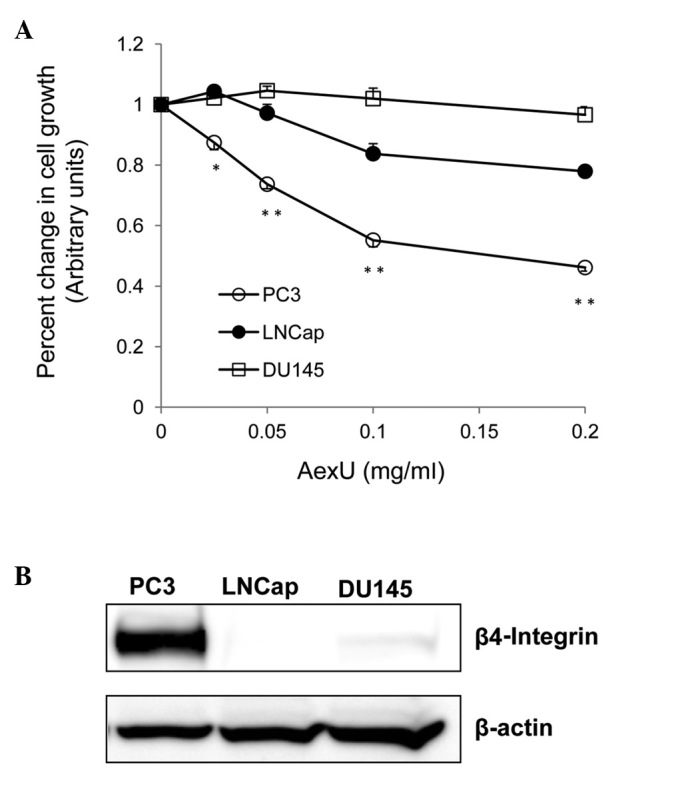
(A) Cytotoxic effects of AexU on the growth of human prostate cancer cells. PC3, LNCaP and DU145 cells were treated with the indicated concentrations of AexU. Following a 24-h incubation period, cell growth was assessed using Cell Counting kit-8. Each data point and bar represent the mean ± SD of triplicate analyses (^**^P<0.01 and ^*^P<0.05 vs. LNCaP and DU145, respectively). (B) β4-integrin expression in human prostate cancer cells. The expression levels of β4-integrin and β-actin in the PC3, LNCaP and DU145 cells were analyzed using western blot analysis.

The cytotoxic effects of AexU on the three human prostate cancer cell lines were examined. AexU exhibited dose-dependent cytotoxic activity in the PC3 cells, whereas there were no significant growth inhibitory effects of AexU on the LNCaP or DU145 cells (Fig. 1A). β4-integrin protein expression levels were examined in these cells using western blot analysis, and abundant β4-integrin expression was identified in the PC3 cells, but not in the LNCaP or DU145 cells (Fig. 1B).

### Inactivation of Akt in PC3 cells following treatment with AexU

**Figure 2:**
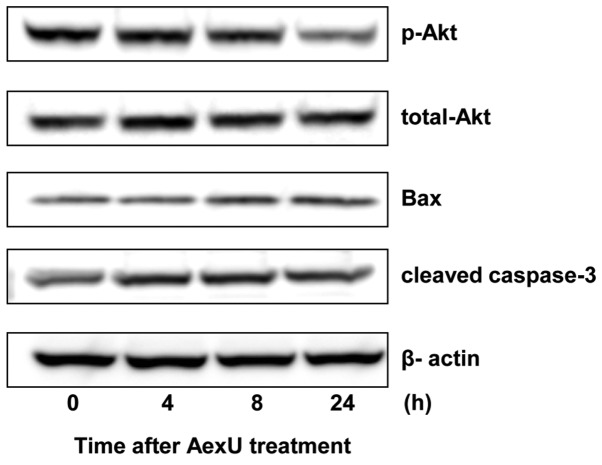
Changes in the expression patterns of key molecules that are involved in the cytotoxic effects of AexU in PC3 cells. The expression levels of phosphorylated (p) and total Akt, Bax, cleaved caspase-3 and β-actin in PC3 cells following treatment with AexU were analyzed using western blot analysis.

Despite the lack of change in the total Akt expression, the phosphorylated Akt in the PC3 cells was downregulated following treatment with AexU (Fig. 2). Furthermore, AexU treatment induced an increase in the expression levels of Bax and caspase-3 in the PC3 cells.

### Chemosensitization of PC3 cells by AexU treatment

**Figure 3:**
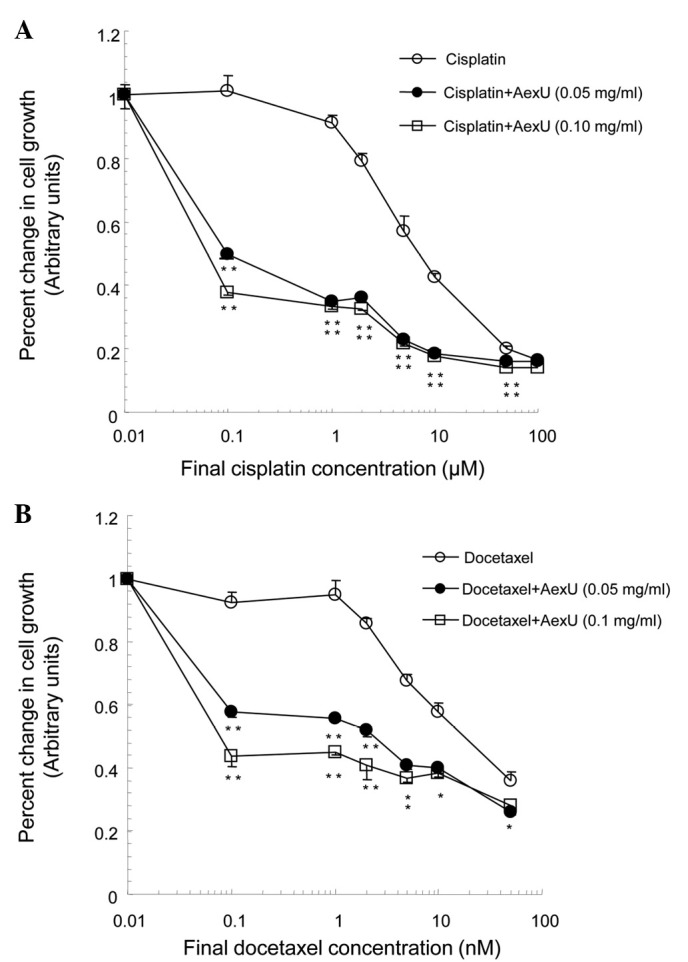
Growth inhibitory effects of AexU in combination with chemotherapeutic agents on PC3 cells. PC3 cells were treated with 0.05 or 0.1 mg/ml AexU in combination with the indicated concentrations of (A) cisplatin or (B) docetaxel. Following a 48-h incubation period, the number of viable cells was assessed using Cell Counting kit-8. Each data point and bar represents the mean ± SD of triplicate analyses (^**^P<0.01 and ^*^P<0.05 vs. controls treated with the chemotherapeutic agent alone).

To determine whether AexU enhances the chemosensitivity of PC3 cells, the cells were treated with various concentrations of cisplatin or docetaxel in combination with AexU. AexU significantly enhanced the sensitivity of the PC3 cells to cisplatin and docetaxel (Fig. 3). The IC_50_ values of the two agents in the PC3 cells following treatment with AexU decreased by ~90% compared with those of the PC3 cells that were treated with either of the chemotherapeutic agents alone.

### Establishment of PC3 sublines transduced with a vector-based shRNA targeting β4-integrin

**Figure 4:**
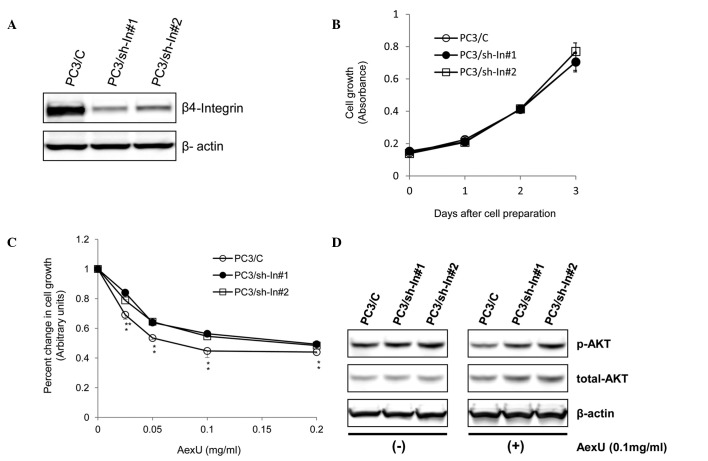
(A) β4-integrin expression in PC3 sublines. The expression levels of β4-integrin and β-actin in the PC3 sublines (PC3/C, control vector-only transfected cell line; PC3/sh-In#1 and PC3/sh-In#2, β4-integrin shRNA-transfected cell lines) were analyzed using western blot analysis. (B) The growth patterns of the PC3 sublines. The proliferation of the PC3 sublines were assessed using Cell Counting kit-8. Each data point and bar represents the mean ± SD of triplicate analyses. (C) The effects of AexU on the growth of the PC3 sublines. The PC3 sublines were treated with the indicated concentrations of AexU. Following 24 h of incubation, cell growth was assessed using Cell Counting kit-8. Each data point and bar represents the mean ± SD of triplicate analyses (^**^P<0.01 and ^*^P<0.05 vs. the PC3/C cells). (D) The changes in the expression patterns of Akt in the PC3 sublines following AexU treatment. The expression levels of phosphorylated (p) and total Akt and β-actin in PC3 sublines prior to and following the treatment with AexU were analyzed using western blot analysis.

To further characterize the association between the cytotoxicity of AexU and the expression of β4-integrin in prostate cancer, the PC3 sublines, PC3/sh-In#1 and PC3/sh-In#2, were established by transfecting the cells with a vector-based shRNA targeting β4-integrin. The expression levels of β4-integrin in the PC3/sh-In#1 and PC3/sh-In#2 cells were markedly inhibited by >50% compared with the negative control vector-transfected sublines (PC3/C) (Fig. 4A).

The growth patterns of the PC3 sublines with and without AexU treatment were investigated. There were no significant differences in the growth between the untreated PC3 sublines (Fig. 4B). However, treatment of the PC3 sublines with AexU revealed that the PC3/sh-In#1 and PC3/sh-In#2 cells were significantly less sensitive to AexU than the PC3/C cells (Fig. 4C). Furthermore, there were no significant differences in the expression levels of either total or phosphorylated Akt among the PC3 sublines, but AexU treatment resulted in a marked inhibition of phosphorylated Akt in the PC3/C cells compared with the PC3/sh-In#1 and PC3/sh-In#2 cells (Fig. 4D).

## Discussion

Prostate cancer is the most common malignancy and the second leading cause of cancer-related mortality in males in Western industrialized countries (18). Although androgen withdrawal remains the only effective therapy for advanced prostate cancer, the majority of patients with advanced disease undergo a progression to castration-resistant prostate cancer (CRPC) within a few years following the initiation of this therapy (19). In recent years, several novel agents have demonstrated a prognostic advantage in males with CRPC and have been introduced into clinical practice (20). However, these improvements have been modest, suggesting the requirement for the development of new agents with alternative mechanisms of action to those of the currently available agents. Therefore, the present study investigated the cytotoxicity of a TTSS effector, AexU, which has been shown to have a therapeutic effect against cancer cells (10) and human CRPC PC3 cells. The present study focused on the involvement of β4-integrin expression on the AexU-mediated antitumor activity.

The study demonstrated the potent dose-dependent cytotoxic activity of AexU against the PC3 cells. Several previous studies have addressed the mechanism of action underlying the cytotoxic effects that are induced by AexU (3,4). Sierra *et al*(4) revealed a characteristic rounded morphology of HeLa cells that were transfected with the AexU gene, which was attributed mainly to the NH2-terminal portion of this toxin. This phenomenon may be explained by the pronounced ADP-ribosyltransferase activity in the NH2-terminal domain of AexU, which was demonstrated to induce the rounded cell morphology by disrupting the function of actin filaments (21), thus interfering with significant host cell functions, including motility, phagocytosis, secretion, proliferation and mitosis (3,4). The actin cytoskeleton in human cancer cells has been reported to be disrupted by AexU, which appears to co-localize with β4-integrin and is more effective in cells overexpressing β4-integrin (10). In fact, in the present study, the sensitivity of the LNCAP and DU145 cells, which express undetectable levels of β4-integrin, was shown to be much lower than that of the PC3 cells, which have an abundant expression of β4-integrin. Consistent with the previous study, this finding suggests that β4-integrin expression is involved in the AexU-mediated cytotoxicity of AexU against cancer cells.

The mechanism by which AexU induces cytotoxic effects in the PC3 cells is worthy of attention. Among the various major signaling pathways that we examined (data not shown), Akt was identified to be markedly inactivated in the PC3 cells following treatment with AexU. To date, there have been several studies that have investigated the changes in host cell signaling elicited by bacterial proteins that are delivered via the TTSS (22,23). However, the present study is the first to characterize the changes in signal transduction that are caused by AexU. The present study subsequently assessed whether AexU treatment altered the apoptosis-related molecules in the PC3 cells and revealed that there was upregulation of Bax and cleaved caspase-3. Sierra *et al*(4) also reported the activation of caspase-3 and caspase-9 in HeLa cells producing the AexU toxin. Collectively, these findings suggest that the cytotoxicity of AexU results from apoptotic cell death via the mitochondrial stress pathway.

To the best of our knowledge, there have not been any previous studies that have evaluated the efficacy of TTSS effectors as anticancer agents, including AexU. Therefore, the antitumor activity of AexU in combination with other agents has not been examined. The present study assessed whether the additional treatment of cells with chemotherapeutic agents, including cisplatin and docetaxel, was able to increase AexU-induced cytotoxicity in the PC3 cells. A markedly enhanced sensitivity of the PC3 cells to the chemotherapeutic agents was observed following treatment with AexU. Considering these findings, it may be worth exploring agents that enhance the cytotoxicity of AexU against cancer cells more effectively than these chemotherapeutic agents.

The present study established PC3 cells in which β4-integrin expression was markedly inhibited by introducing an expression plasmid. The plasmid contained shRNA targeted against the β4-integrin gene in order to elucidate the role of β4-integrin in AexU-induced cytotoxicity in the PC3 cells. Despite the fact that no significant differences in proliferation were observed between the PC3 sublines without AexU, the suppression of the β4-integrin protein resulted in a decrease in the sensitivity of the PC3 cells to AexU. Furthermore, the activation of Akt following the treatment of the control PC3 cells, but not the β4-integrin knockdown PC3 cells, appeared to be markedly inhibited. These findings suggest that AexU-induced cytotoxicity in the PC3 cells is mediated, at least in part, by the inactivation of Akt signaling regulated by the expression of the β4-integrin protein.

In conclusion, a TTSS effector, AexU, was shown to exert potent cytotoxic activity against PC3 cells through the inactivation of the Akt signaling pathway. This effect may be significantly enhanced by an additional treatment with chemotherapeutic agents. Furthermore, the β4-integrin protein appears to be involved in the mechanism underlying AexU-induced apoptotic cell death. Collectively, the results of the present study suggest that AexU-based therapy may be a promising novel strategy for CRPC. However, additional experiments, particularly *in vivo* studies, are required to further investigate the potential of AexU as a therapeutic agent for this condition.
